# Rapid Assessment of Avoidable Visual Impairment in Two Coastal Districts of Eastern India for Determining Effective Coverage: A Cross-Sectional Study

**DOI:** 10.18502/jovr.v18i2.13185

**Published:** 2023-04-19

**Authors:** Amit Bhardwaj, Praveen Vashist, Suraj Singh Senjam, Vivek Gupta, Noopur Gupta, Souvik Manna

**Affiliations:** ^1^Community Ophthalmology, Dr. Rajendra Prasad Centre for Ophthalmic Sciences, All India Institute of Medical Sciences, New Delhi, India; ^2^Department of Ophthalmology, Dr. Rajendra Prasad Centre for Ophthalmic Sciences, All India Institute of Medical Sciences, New Delhi, India

**Keywords:** Blindness, Cataract, Refractive Error

## Abstract

**Purpose:**

To measure the prevalence and causes of visual impairment (VI) among the 40+ age population in two coastal districts of India and to determine the levels of effective cataract surgical coverage (eCSC) and effective refractive error coverage (eREC) in the study population.

**Methods:**

A cross-sectional study was done on 4200 people chosen using cluster sampling in two coastal districts of Odisha, an eastern state in India. A team consisting of trained optometrists and social workers conducted the ocular examination which included unaided, pinhole, and aided visual acuity assessments followed by examination of the anterior segment and lens.

**Results:**

Overall, 3745 (89.2%) participants were examined from 60 study clusters, 30 in each district. Among those examined, 1677 (44.8%) were men, 2554 (68.2%) were educated and number? (17.8%) used distance spectacles during the survey. The prevalence of VI adjusted for age and gender was 12.77% (95% CI 11.85–13.69%). Multiple logistic regression showed that older age (OR 3.1; 95% CI 2.0–4.7) and urban residence (OR 1.2; 95% CI 1.0–1.6) were associated with VI. Being educated (OR 0.4; 95% CI 0.3–0.6) and using glasses (OR 0.3; 95% CI 0.5–0.2) were found to provide protection; therefore, resulting in lower instances of VI. Cataract (62.7%) and uncorrected refractive errors (27.1%) were the two main causes of VI. The eCSC was 35.1%, the eREC for distance was 40.0%, and the eREC for near was 35.7%.

**Conclusion:**

VI remains a challenge in Odisha, as the prevalence is high and the surgical coverage is poor. Nearly 90% of VI is avoidable indicating that targeted interventions are required to address this problem.

##  INTRODUCTION

Vision impairment (VI), including blindness, is an alarming global public health issue with a disproportionately larger prevalence in low- and middle-income countries (LMICs). As per the recent WHO estimates, globally, approximately 2.2 billion people have near and distance VI of which almost 50% need either prevention or treatment measures.^[1]^ In October 2019, WHO launched the first World Report on Vision to draw attention to the increasing need for eye care.^[2]^

India has the second largest population in the world with nearly 4.8 million of the population suffering from blindness and 74 million experiencing VI.^[3]^ Cataract and uncorrected refractive errors (URE) are the two commonest reasons for VI in India.^[3]^ In a country like India, where the population is diverse and heterogeneous, it is imperative to have regionally representative, valid, and robust public health data so that appropriate public health strategies can be planned at the local level. Rapid assessment of VI (RAVI) surveys are less expensive and less time-consuming as compared to detailed and resource-intensive epidemiological studies. For tracking progress at country and regional levels, RAVI surveys are needed at periodic intervals to assess baseline status and progress toward targets. Effective refractive error coverage (eREC) and effective cataract surgical coverage (eCSC) are also tracer indicators that gauge the eye care scenario in a country.^[4]^ The global targets include a 30% increase in eCSC and a 40% increase in eREC, by 2030.[4, 5]

RAVI surveys have been completed in many areas of southern, western, central, and northern India; however, relevant data on the magnitude of the VI burden, especially in the remote underprivileged population from Eastern India is missing. A RAVI study was conducted in the population aged 40+ in two coastal districts of Odisha with the purpose of assessing the magnitude and causes of VI in the two districts and to determine the levels of eCSC and eREC in the study population.

##  METHODS 

Two coastal districts were selected in the state of Odisha: Ganjam which is predominantly rural and Khordha which is predominantly urban.[6, 7] The sample size of 2100 per district was selected using a prevalence of 15% VI in the 40+ age group, a relative precision of 15%, confidence interval (CI) of 95%, 1.75 design effect (cluster size 70), and a nonresponse rate of 20%.^[8]^ The study protocol was reviewed and approved by Institute Ethics Committee of All India Institute of Medical Sciences, New Delhi, India under the approval number IEC-562/02.12.2016,RP/8/2016, OP-10/06.03.2020. In addition, the protocol of the study complied with the guidelines for human studies and the World Medical Association Declaration of Helsinki.

Sampling was done in multiple stages using cluster random techniques. In urban regions of India, the district is divided into municipal wards, whereas in the rural areas the administrative divisions are villages. A list of all the municipal wards and villages in the district was obtained from the Census Office, India. In the first stage of sampling, all the subdivisions were selected, and within each subdivision, a list of 30 PSUs (primary sampling units) comprising of urban wards and villages was chosen based on the probability proportionate to size (PPS) techniques. The PSUs had a maximum population size of 2000. If a village or ward had a population greater than 2000, it was divided into smaller PSUs of size 2000, each of which was independently entered into the sampling frame. Within each PSU, the selection of households involved a compact segment sampling technique in which the selected PSU was divided into multiple segments of 300–500 people and one such segment was chosen randomly by a draw of chits. In the selected segment, the survey proceeded from one corner and all contiguous houses were visited until 70 people were enumerated. By covering a total of 30 such segments, the target sample of 2100 in each district was achieved.

The sample population comprised of all those who were aged 40+ and were habitual residents of the selected districts (living in the area for at least six months). Two teams were dispatched to visit 30 clusters in each district. Before the commencement of the study, a two-day training was given to the team regarding standard study protocols, method of cluster selection and coding, enumeration methods, clinical examination, and barrier information. Inter-observer variations among the optometrists for clinical diagnosis, distant and near vision examinations were checked in the clinic and community. The inter-observer agreement was good (kappa > 0.8) for all survey procedures among optometrists as each team consisted of members from local areas who were helpful in overcoming any language or cultural barriers.

The survey was conducted using the standard RAVI questionnaire. It captured data on the avoidable reasons for blindness and VI in people aged 40+. This questionnaire was modified from the standard RAAB (Rapid Assessment of Avoidable Blindness), and had extra sections for near vision, use of glasses, and unaided visual acuity.

Presenting binocular near vision was measured using a simplified “E” chart having N60 and N6 optotypes with five letters in one line. The procedure was performed at a distance of 40 cm, which was ensured by using a headband attached to a rope 40 cm in length. Near vision was calculated first using the N60 optotype and then using the N6 optotype. The criterion for determining the category of vision at a certain level was selection of four correct letters out of five from the simplified “E” chart.

**Figure 1:**
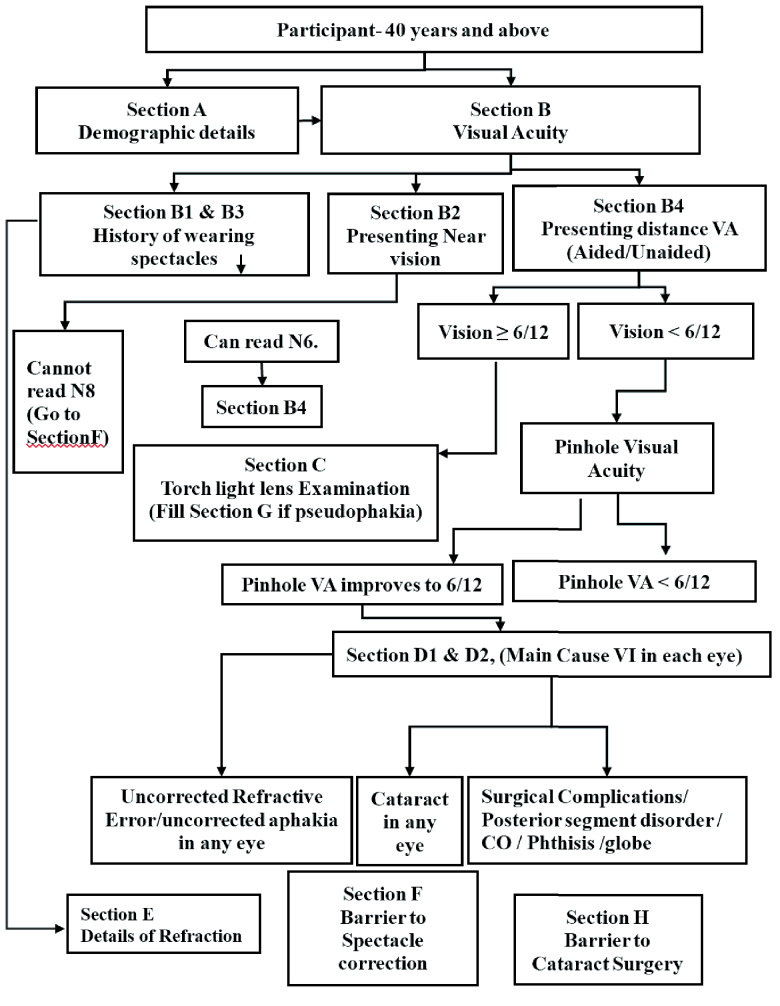
Examination protocol of the RAAVI Study in Khordha and Ganjam districts of Odisha.

Distance visual acuity (VA) was tested utilizing tumbling “E” charts both with and without spectacles. VA was examined with “E” Snellen optotypes of different sizes for VA of 6/12, 6/18, and 6/60 at 6 m. The criterion for measuring vision at any of the levels was the selection of four correct answers consecutively, or four correct out of five tumbles. If the person wore spectacles for distance vision, the pinhole was placed in front of the spectacles. The lens assessment was done in an undilated pupil with a pen torch [Figure 1].

The primary outcome measure was VI, which was defined as presenting visual acuity (PVA) < 6/12 according to the International Classification of Diseases (ICD-10 as revised by WHO)).^[9]^

The main cause of PVA < 6/12 was separately ascertained for each eye, and the more avoidable cause was taken as the diagnosis for each person. In cases with multiple causes for VI, the disease that was more preventable/amenable to treatment to achieve VA ≥ 6/12 was considered as the principal cause. The data entry was done in specially designed Epi-data 3.1-based database with all checks in place for validation and data consistency. Double data entry was done to minimize errors and data cleaning was done to remove all inconsistent findings and outliers. Data analysis was done using the Stata 15.1 software package (Stata Corp., College Station, Texas, USA). Age and gender disaggregated prevalence of VI along with 95% CI were calculated. Univariate and multivariate analyses were done to find factors associated with VI. The sample prevalence (unstandardized) was directly standardized using the age-sex distribution of the combined population of the two districts, using Stata software. Association of VI with age, gender, education, and locality was checked by using multiple logistic regression analysis.

##  RESULTS

Overall, 4200 individuals aged 40+ were enumerated, of whom 3745 (89.2%) were examined. The number of males was 1677 (44.8%) and that of females was 2068 (55.2%). The response rate was better among females (91.6%) than males (86.4%), as far as clinical examination was concerned. Out of the 455 not examined, 441 were either not available or were unable to communicate and 14 refused to undergo examination. There was no significant difference between the socio-demographic profile of the sample and target population (Odisha state) (*P* = 0.57) [Table 1].

**Table 1:** Socio-demographic profile of 40+ sample population in Khordha and Ganjam districts compared with Odisha state.


**Variable**	<@orange**Sample**			<@orange**Odisha**		
	**Male (%)**	**Female (%)**	**Total (%)**	**Male (%)**	**Female (%)**	**Total (%)**
**Age groups (yr)**						
40–49	449 (26.7)	705 (34.1)	1154 (30.8)	26,00,286 (41.3)	24,25,218 (40.1)	50,25,504 (40.7)
50–59	438 (26.1)	550 (26.6)	988 (26.4)	17,01,815 (27.0)	16,29,717 (26.9)	33,31,532 (26.9)
60–69	440 (26.2)	510 (24.7)	950 (25.4)	12,25,484 (19.5)	12,28,102 (20.3)	24,53,586 (19.9)
70–79	260 (15.5)	240 (11.6)	500 (13.4)	5,63,929 (8.9)	5,68,941 (9.4)	11,32,870 (9.2)
≥ 80	90 (5.4)	63 (3.0)	153 (4.1)	2,04,857 (3.3)	1,93,135 (3.2)	3,97,992 (3.2)
Total	1677	2068	3745	62,96,371	60,45,113	1,23,41,484
**Education **			
Illiterate	274 (16.3)	917 (44.3)	1191 (31.8)	18,76,376 (29.8)	36,96,032 (61.2)	55,72,408 (45.2)
Up to 4 ${}^{th}$	380 (22.7)	548 (26.5)	928 (24.8)	12,06,268 (19.2)	8,34,639 (13.8)	20,40,907 (16.6)
5 ${}^{th}$ –9 ${}^{th}$ pass	419 (24.9)	351 (16.9)	770 (20.6)	18,98,155 (30.2)	10,83,901 (17.9)	29,82,056 (24.2)
> 10 ${}^{th}$ pass	604 (36.0)	252 (12.2)	856 (22.9)	13,06,345 (20.8)	4,24,874 (7.0)	17,31,219 (14.0)
**Total **	1677	2068	3745	62,87,144	60,39,446	1,23,26,590
	
	

**Table 2:** Prevalence of visual impairment and blindness among 40+ population in Khordha and Ganjam districts of Odisha.


	<@orange**Combined % (95% CI)**					**Unstandardized prevalence (%)**	**Standardized prevalence (%)**
	Definition	**Male (** ***N*** ** = 1677)**		**Female (** ***N*** ** = 2068)**		**Total**	
Blind	PVA < 3/60 in BE*	27	1.61 (1.00–2.21)	27	1.30 (0.81–1.79)	54	1.44 (1.06–1.95)	1.14 (0.84–1.45)
SVI	PVA < 6/60 – 3/60 in BE*	23	1.37 (0.81–1.93)	30	1.45 (0.93–1.96)	53	1.41 (1.15–1.73)	1.13 (0.83–1.43)
MVI	PVA < 6/18 – 6/60 in BE*	107	6.38 (5.20–7.55)	113	5.46 (4.48–6.44)	220	5.87 (5.10–6.75)	4.84 (4.22–5.46)
Mild VI	PVA < 6/12 – 6/18 in BE*	117	6.97 (5.75–8.19)	135	6.52 (5.46–7.59)	252	6.72 (5.74–7.86)	5.64 (4.96–6.33)
VI	PVA < 6/12 in BE*	274	16.33 (14.56–18.11)	305	14.74 (13.21–16.27)	579	15.46 (14.06–16.96)	12.77 (11.85–13.69)
	
	
*PVA, presenting visual acuity; BE, better eye with available correction or with best correction or pinhole (BCVA or PINVA); SVI, severe visual impairment; mvi, moderate visual impairment; VI, visual impairment; CI, confidence interval.								

**Table 3:** Age-wise prevalence of visual impairment and uncorrected refractive error among 40+ population in Khordha and Ganjam districts of Odisha.


**Age group (yr)**	**Prevalence of VI (PVA < 6/12 in better eye) (%)**								**Prevalence of uncorrected refractive error (%) with 95% CI**
	**Male**	**Prev (%)**	**Female **	**Prev (%)**	**Total**	**Prev (%)**	**Odds ratio**	***P*** **-value**	
40–49	11	2.4	20	2.8	31	2.7	1	0.000	3.1 (2.19–4.29)
50–59	44	10.0	37	6.7	81	8.2	3.28 (2.15–5.00)	0.000	7.7 (6.11–9.53)
60–69	83	18.9	106	20.8	189	19.9	9.06 (6.13–13.38)	0.000	14.4 (12.25–16.82)
70–79	82	31.5	99	41.2	181	36.2	20.38 (13.65–30.42)	0.000	14.2 (11.26–17.57)
80+	54	60.0	43	68.3	97	63.4	62.75 (38.62–101.95)	0.000	15.0 (9.77–21.70)
Total	274	16.3	305	14.7	579	15.5		9.2 (8.25–10.13)
	
	
*Prev, prevalence; VI, visual impairment; CI, confidence interval; PVA, presenting visual acuity									

**Table 4:** Cataract surgical coverage and effective coverage among 40+ population in Khordha and Ganjam districts of Odisha.


**CSC (persons)**	**Male (%)**	**Female (%)**	**Total (%)**
PinVA < 3/60	88.29	88.11	88.19
PinVA < 6/60	84.30	79.88	81.72
PinVA < 6/18	66.67	59.83	62.74
PinVA < 6/12	49.08	46.04	47.40
eCSC	33.70	36.09	35.02
eREC for distance	38.10	41.91	40.08
eREC for near	40.01	32.83	35.65
	
	
*CSC, cataract surgical coverage; PinVA, pinhole visual acuity; eCSC, effective cataract surgical coverage; eERC, effective refractive error coverage			

**Table 5:** Multiple logistic regression analysis for finding determinants of visual impairment in Khordha and Ganjam districts of Odisha.


**Variable**	**Odds ratio**	**95% Confidence Interval**	**z-value**	***P*** **-value**
**Age groups (yr)**				
40–49	1		
50–59	3.07	2.00–4.69	5.16	< 0.001
60–69	7.71	5.16–11.51	9.97	< 0.001
70–79	16.48	10.86–25.03	13.15	< 0.001
≥ 80	51.19	30.61–85.60	15.00	< 0.001
**Gender**		
Male	1		
Female	0.79	0.64–0.99	–1.98	0.048
**Locality**		
Rural	1		
Urban	1.26	1.02–1.57	2.18	0.03
**Education **		
Illiterate	1		
Up to 4 ${}^{th}$	0.62	0.48–0.81	–3.53	< 0.001
5 ${}^{th}$ –9 ${}^{th}$ pass	0.62	0.45–0.84	–3.09	0.002
> 10 ${}^{th}$ pass	0.39	0.27–0.56	–5.04	< 0.001
**Use of distance glasses**				
No	1		
Yes	0.31	0.46–0.21	–5.84	< 0.001
	
	
*RAAVI, rapid assessment of avoidable visual impairment; VA, visual acuity; VI, visual impairment; CO, corneal opacity.				

**Figure 2:**
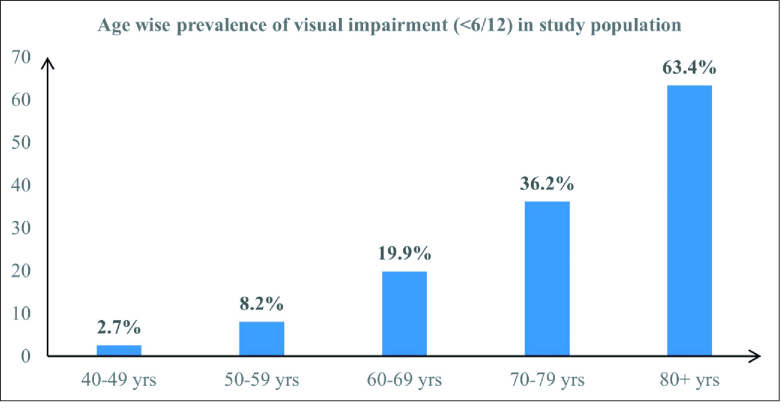
Age-wise prevalence of visual impairment among 40+ population in Khordha and Ganjam districts of Odisha.

**Figure 3:**
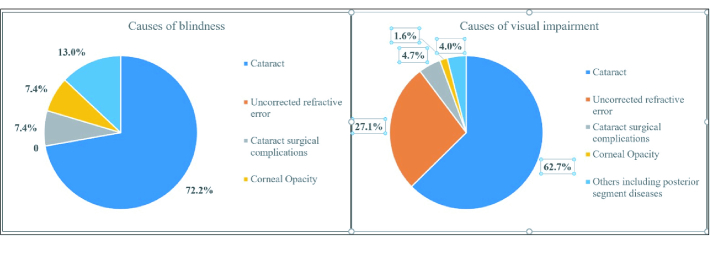
Causes of blindness and visual impairment (PVA < 6/12 in better eye) among 40+ population in Khordha and Ganjam districts of Odisha.

The age and sex standardized prevalence of VI including blindness was 12.77% (95% C.I. 11.85–13.69). Although the prevalence of VI (including blindness) was higher in males (16.33%, 95% CI: 14.56–18.11) than females (14.74%, 95% CI:13.21–16.27), the difference was not statistically significant (*P* = 0.181). The standardized prevalence of moderate VI (MVI: presenting visual acuity < 6/18 to 6/60 in the better eye) and severe VI (SVI: presenting visual acuity < 6/60 to 3/60 in the better eye) in study subjects was 4.84% and 1.13%, respectively. The blindness prevalence in the study population was 1.14% (95% C.I. 0.84–1.45) [Table 2].

The prevalence of VI and URE exhibited a rising trend with age. Maximum VI (including blindness) was seen in females above the age of 80 years (68.3%) [Figure 2 & Table 3]. Out of the 3745 study participants, 343 (9.2%) had URE. Another 155 (4.1%) had corrected refractive errors (RE) giving a total of 498 individuals with any form of RE (13.3%). Prevalence of URE among males was 9.5% (95% CI: 8.12–10.99) and among females was 8.9% (95% CI: 7.71–10.21); the difference was not significant (*P* = 0.538) [Table 3]. The prevalence of VI in rural participants was 16%, which was higher than that of urban participants (14.6%). Cataract was the single most important cause of blindness (72.2%) and VI (62.7%) in this region [Figure 3].

A total of 536 cataract surgeries were reported among people from the study population. More females reported surgery (288) as compared to males (248). The proportion of surgeries with an intraocular lens (IOL) implant was 94.0%. Out of the 377 persons (536 eyes) who underwent cataract surgery, 159 were bilaterally operated while 218 had undergone unilateral cataract surgery. It was observed that the majority of unilateral and bilateral cataract surgeries were performed in people aged 60 years and above.

When visual outcomes were assessed in the operated eye, it was seen that 355 eyes (66.2%) had very good outcomes ( ≥ 6/12), 54 (10.1%) had good outcomes ( < 6/12 to 6/18), 57 (10.6%) had borderline outcomes ( < 6/18 to 6/60), and 70 (13.1%) had poor outcomes ( < 6/60). Poor outcomes were caused by operative complications in 50 (71.4%) eyes, ocular comorbidity in 18 (25.7%), and RE in 2 (2.9%) eyes. Nearly half of the surgeries, that is, 253 (47.2%) were performed in the last five years. The visual outcomes were better among surgeries reported in the last five years as compared to surgeries performed before that, and the difference was statistically significant (*P* = 0.014). The CSC (Cataract Surgical Coverage) in two districts of Odisha for 40+ population was 47.4% by 6/12 cut-off and 88.19% for pinhole vision < 3/60. The eCSC was 35.1%, eREC for distance was 40.0%, and eREC for near was 35.7% for the same population [Table 4].

Out of the 536 cataract surgeries, 300 (55.9%) took place in non-governmental organizations (NGO)/private sector as compared to 236 (44.0%) in the public sector, and 287 (53.5%) of the surgeries were paid irrespective of the place of surgery.

The usage of distance glasses was reported by only 518 (13.8%) study participants (males 265 [15.8%] and females 253 [12.2%]). Similarly, near glasses were being used by only 666 (17.8%) participants (males 364 [21.7%] and females 302 [14.6%]). Out of a total of 735 participants who received refraction services, 505 (68.7%) had their last refraction more than two years back. Most refractions, that is, 557 (75.8%) took place in the NGO/private sector (males 74.7% and females 77.3%). The majority (89.7%) of participants reported having to pay for their glasses irrespective of place of refraction.

Barriers to not wearing glasses among those who were identified as having URE/presbyopia or uncorrected aphakia were assessed. The most common barriers were need not felt (57.5%), financial constraints (13.8%), uncomfortable glasses (8.2%), various local reasons like no one to accompany them, and other personal preoccupations (11.0%) in addition to lack of awareness (5.0%).

Multiple logistic regression was done to find determinants of VI in the study population. The odds of VI increased significantly in higher ages and were greater among the urban population (OR 1.2; 95% CI 1.0–1.6). Being educated (OR 0.4; 95% CI 0.3–0.6) and use of glasses (OR 0.3; 95% CI 0.5–0.2) were protective. All these risk factors are well-established as associated with VI [Table 5].

##  DISCUSSION

The current study utilized the novel RAVI methodology to determine the prevalence and causes of VI in two districts of Odisha, India. According to the National Blindness Survey (NBS), the prevalence of blindness in the 50+ age population in India was 1.99% and cataract (66.2%) was the most important cause of blindness followed by corneal opacity (8.2%).^[10]^

In the current study, the age-sex-adjusted prevalence of blindness was 1.14%; MVI was 4.84%, and VI was 12.77% among the 40+ age population in Odisha. The NBS was conducted among the 50+ age population in one district of Odisha (Nayagarh) and the prevalence of blindness and MVI reported were 1.77% and 13.4%, respectively.[3, 10] Although the prevalence figures in the current study are lower as compared to NBS, it might be due to the lower age group (40+) and other differences among the survey participants. Despite this, the VI due to cataract and RE needs to be managed promptly as the study clearly highlights poor coverage for cataract and RE services in Odisha.

The study demonstrated that the risk of VI increased significantly with age, which is being corroborated by numerous studies done in both South and North India.[8, 11, 12] A previous study on blindness had reported that females were at 1.41 times higher risk of blindness in urban areas and 1.51 times in rural areas, compared to their male counterparts.^[13]^ However, no significant difference was observed by gender in the current study. Cataract is a major cause of VI and blindness in the current study. These findings are similar to the findings from South India.^[14]^ Cataract and REs combined contributed to > 90% of VI both of which are amenable to treatment as compared to the other causes. Cataract surgery has been identified as surgical intervention that costs < $200 per disability-adjusted life years averted.^[15]^

The CSC of Odisha for the 40+ age population was determined as 47.4%. In the NBS, a CSC (persons) of 50.0% at a VA cut-off of 6/18 was reported in Nayagarh, Odisha.^[3]^ Another RAAB study by SightSavers in the Kalahandi district of Odisha reported a CSC of 45.5%.^[16]^ In both studies, males had higher CSC than females, similar to the findings of the current study. A study from rural Northern India reported higher CSC in females than males, with CSC of 43.2% at 6/60 cut-off.^[17]^ Lower CSC and thereby higher prevalence of cataract and VI was observed in rural areas in many countries.[18, 19] The eCSC determined in the current study was 35.02% and it was higher in females as compared to males. Very few studies have reported eCSC from the Indian subcontinent. A preliminary analysis (unpublished) of 47 population-based surveys from 11 countries revealed a significant range in eCSC between countries, from 2.8% to 88.5%.^[4]^ Data from repeated population-based surveys within four LMICs revealed an average annual percentage point increase in eCSC of 1.1% (range = 0.8% - 1.4%). In addition, gender inequities in eCSC have been reported: it is estimated that globally, women were 1.21 times more susceptible to having cataract VI as compared to men, and the mean level of inequality amongst women in eCSC is 4.6%.[20, 21]

In order to know the exact number of people with VI due to RE, uncorrected visual acuity needs to be measured, that is, without spectacles or contact lenses.^[4]^ The current study employed this methodology, and the prevalence found was 9.2%. In the current study, the eREC for distance was 40.1%, while for the near vision it was 35.6%. Rates of eREC for near is lower than 20% in sub-Saharan Africa, while the same figures in North America are reported to be higher than 90%.^[22]^

The impact of REs is manifold and includes loss of livelihood, schooling, and financial resources.^[23]^ Estimates of global economic burden of distant VI due to URE is huge (US$ 202 billion).^[24]^ Globally, nearly 800 million people suffer from distance VI (i.e., myopia and hypermetropia) or near VI (i.e., presbyopia) who need just a pair of spectacles, while another 100 million persons have moderate-to-severe distance VI or blindness that is amenable by cataract surgery.^[25]^ The sustainability of programs for treatment of cataract and RE need huge expenditure in terms of equipment, manpower, and spectacles. Hence, sale of customized spectacles can also be explored as an alternative source of revenue by hospitals that want to scale-up their cataract surgical and refractive services, as envisioned by the Vision 2020 Right to Sight Initiative. The Government of India launched the National Program for Control of Blindness and Visual Impairment (NPCB&VI) in 1976 and it currently has the provision of free services for cataract and other subspecialties; however, with the increase in the number of RE, there is a need to introduce a provision of subsidized/free spectacles also into the program to alleviate some of the additional costs ensued.^[26]^ Generation of demand for services and addressing various barriers for accessing those services is necessary to scale-up the provision of cataract surgical and refractive error services to the population.

The WHO has given targets for achieving universal eye health coverage (UHC) by 2030, that is, a 30% point increase in eCSC and 40% increase in eREC from baseline.^[4]^ This means that Odisha needs to achieve an eCSC and eREC of 65.0% and 80.1%, respectively, by 2030, from the baseline figures reported in the current study. This can be possible only if the management of VI is prioritized in Odisha in a systematic way. Shortage of trained human resources and resource constraints are always bottlenecks for such ambitious targets. The National Program for Control of Blindness and Visual Impairment (NPCBVI) can provide effective RE and cataract surgical services free of cost or at subsidized rates, which can be incorporated in the health insurance packages available to the people.

The current RAAVI study has a few limitations. First, it is not adequately powered to determine the prevalence of blindness. The sample size was deduced based on the prevalence of VI in previous studies and would only provide accurate estimation of the prevalence of VI. Second, the findings of the study cannot be extrapolated to other districts of Odisha, and separate surveys need to be conducted in each of the districts to accurately gauge the magnitude of the problem in the entire state. The burden of VI needs prompt attention in Odisha, majority of which is caused by cataract and RE. The findings suggest that the absolute number of people susceptible to avoidable blindness is enormous, and free or subsidized cataract and RE services is the need of the hour. It is hoped that this baseline study from Odisha will be instrumental in planning eye care services in the state in the future. Some of the major recommendations from the current study include, graded scaling up of cataract and RE services over the next decade to achieve UHC targets by 2030, reducing cost of services by incorporating services in insurance packages and prioritizing vulnerable individuals like the elderly, illiterate, and urban poor. RAAVI studies need to be conducted in all the districts of Odisha, and district-specific interventions need to be planned.

##  Ethical Considerations

The study protocol was reviewed and approved by Institute Ethics Committee of All India Institute of Medical Sciences, New Delhi, India under the approval number IEC-562/02.12.2016,RP/8/2016, OP-10/06.03.2020. In addition, the protocol of the study complied with the guidelines for human studies and the World Medical Association Declaration of Helsinki. A participant information sheet (PIS) in local language Odia was given to each participant. In case of illiterate or visually impaired participants, the PIS was read out to the participant. Written consent was obtained from each participant before they were included in the study. In case of illiterate persons, left thumb impression was obtained. Participants who were identified with treatable or curable conditions were provided referral services to the nearest secondary/tertiary eye hospital.

##  Financial Support and Sponsorship

Financial support for this study was provided by CBM, India. Consent for publication was taken according to the ICMJE recommendations for protection of research participants.

##  Conflicts of Interest

None.
